# Retirement Migration and Precarity: Material and Social Aspects

**DOI:** 10.1093/geroni/igab046.1714

**Published:** 2021-12-17

**Authors:** Marion Repetti, Toni Calasanti

**Affiliations:** 1 University of Applied Sciences and Arts Western Switzerland HES·SO // Valais-Wallis, Sierre, Valais, Switzerland; 2 VIrginia Tech, Blacksburg, Virginia, United States

## Abstract

Discussions of precarity in later life have tended to focus on the uncertainties of material resources, and the feelings of anxiety that this evokes (e.g., Lain et al. 2019) as some older people thus face the risk of being excluded from the broader society. Although scholars often point to inequalities, such as those based on class and gender, as having an influence on the likelihood of older people experiencing such precarity, ageism is considered only to the extent that it can exacerbate the impact of these statuses through, for instance, labor market experiences. Here, we expand upon the impact of ageism on the social aspects of precarity: the loss of recognition and respect as a person that is at the core of social bonds. Drawing on qualitative interviews we have conducted among Swiss, British, and U.S. older people who migrated to cheaper countries in retirement, we demonstrate that ageism can influence precarity regardless of classes. We find that even among wealthier older migrants, who otherwise might fit the image of the retiree seeking an active lifestyle in a sunny location, the attempt to escape the devaluation heaped upon older people in their original country plays an important role. In their new countries, retired migrants of all classes felt that they were valued and part of a community, and this differed from the ageism in their home countries. We thus argue that ageism be considered in future analyses of precarity in later life.

